# Plasma total cell-free DNA (cfDNA) is a surrogate biomarker for tumour burden and a prognostic biomarker for survival in metastatic melanoma patients

**DOI:** 10.1016/j.ejca.2017.10.029

**Published:** 2018-01

**Authors:** S. Valpione, G. Gremel, P. Mundra, P. Middlehurst, E. Galvani, M.R. Girotti, R.J. Lee, G. Garner, N. Dhomen, P.C. Lorigan, R. Marais

**Affiliations:** a Molecular Oncology Group, Cancer Research UK Manchester Institute, The University of Manchester, Wilmslow Road, Manchester, M20 4GJ, UK; b Christie NHS Foundation Trust, Wilmslow Road, Manchester, M20 4BX, UK

**Keywords:** Melanoma, Total circulating cell-free DNA, Tumour burden, Prognostic biomarker

## Abstract

**Introduction:**

Tumour burden is a prognostic biomarker in metastatic melanoma. However, tumour burden is difficult to measure and there are currently no reliable surrogate biomarkers to easily and reliably determine it. The aim of this study was to assess the potential of plasma total cell free DNA as biomarker of tumour burden and prognosis in metastatic melanoma patients.

**Materials and methods:**

A prospective biomarker cohort study for total plasma circulating cell-free DNA (cfDNA) concentration was performed in 43 metastatic melanoma patients. For 38 patients, paired blood collections and scan assessments were available before treatment and at first response evaluation. Tumour burden was calculated as the sum of volumes from three-dimensional radiological measurements of all metastatic lesions in individual patients.

**Results:**

Baseline cfDNA concentration correlated with pre-treatment tumour burden (ρ = 0.52, *P* < 0.001). Baseline cfDNA levels correlated significantly with hazard of death and overall survival, and a cut off value of 89 pg/μl identified two distinct prognostic groups (HR = 2.22 for high cfDNA, *P* = 0.004). Patients with cfDNA ≥89 pg/μl had shorter OS (10.0 versus 22.7 months, *P* = 0.009; HR = 2.22 for high cfDNA, *P* = 0.004) and the significance was maintained when compared with lactic dehydrogenase (LDH) in a multivariate analysis. We also found a correlation between the changes of cfDNA and treatment-related changes in tumour burden (ρ = 0.49, *P* = 0.002). In addition, the ratio between baseline cfDNA and tumour burden was prognostic (HR = 2.7 for cfDNA/tumour volume ≥8 pg/(μl*cm^3^), *P* = 0.024).

**Conclusions:**

We have demonstrated that cfDNA is a surrogate marker of tumour burden in metastatic melanoma patients, and that it is prognostic for overall survival.

## Highlights

• A prospective biomarker cohort study for total circulating cell-free DNA (cfDNA) in plasma was performed in metastatic melanoma patients. Paired blood collections and scan assessments were studied. CfDNA was measured by means of droplet digital Polymerase Chain Reaction and tumour burden was calculated as the sum of volumes from three-dimensional radiological measurements of all metastatic lesions in individual patients.
• Baseline plasma cfDNA level correlates to tumour volume.
• Plasma cfDNA level and is a surrogate biomarker for tumour burden and tumour burden changes after treatment.
• Plasma cfDNA level is a prognostic marker for survival in metastatic melanoma patients.

**Key Message**: Plasma cfDNA level correlates to tumour volume and is a surrogate biomarker for tumour burden and a prognostic marker for survival in metastatic melanoma patients.

## 1 Introduction

Circulating cell-free DNA (cfDNA) is short fragment (usually 130–180 base pairs) double stranded DNA that is present in blood and other body fluids [1], [2], [3], [4]. Its origin is thought to be mainly from apoptotic or necrotic cell death, although active release mechanisms have also been suggested [5], [6]. Increased levels of cfDNA in the blood are frequently observed in cancer patients and in some settings increased cfDNA is an adverse prognostic factor [7]. However, tumours are not the sole source of cfDNA, and increased levels are also linked to impaired renal clearance and production of white blood cells (WBC) [8], [9]. Moreover, the mechanisms of cfDNA release are poorly understood, and their prognostic value and relationship to tumour burden are controversial [10]. In particular, the correlation between tumour volume and cfDNA is still under study [11].

One explanation for the current lack of evidence to directly correlate tumour burden and cfDNA levels is that precise evaluations of tumour burden are not routinely performed. This is because assessing tumour burden in individual patients is demanding and requires time-consuming procedures to measure all metastatic lesions. Metastatic load in melanoma is considered an important prognostic and predictive factor in melanoma and surrogate biomarkers are currently used for clinical purposes, including the number of metastatic sites and Response Evaluation Criteria in Solid Tumours (RECIST) marker lesion measurement [12], [13]. However, the current version (RECIST 1.1) relies on mono-dimensional measurements of no more than 2 lesions per organ and a maximum of 5 lesions selected at the discretion of the radiologist, therefore is subject to interpretation bias as demonstrated by the significant differences often observed between investigators' and central review's assessments in clinical trials [14], [15], [16]. As a consequence, RECIST 1.1 is a poor tool with which to investigate the relationship between cfDNA and tumour burden.

One of the most powerful uses of cfDNA is related to the identification of tumour-specific mutations that are derived from the cancer cells. The analysis of this circulating tumour DNA (ctDNA) allows application of liquid biopsies for personalised strategies [17], [18], [19], [20]. Critically however, a clear link between cfDNA or ct DNA and tumour burden has not been established, and routine analysis of ctDNA is often unfeasiblebecause it requires information on the mutational landscape of the tumour.

In the present study, we examined the relationship between cfDNA, ctDNA and tumour burden in patients with metastatic melanoma. We used computed tomography (CT) and/or magnetic resonance imaging (MRI) scans to determine the total tumour burden in the patients and then compared this to cfDNA levels. Intriguingly, we did not find a correlation between ctDNA and cfDNA, but did find a correlation between cfDNA and tumour burden. Our study shows the potential of cfDNA as biomarker of tumour burden in metastatic melanoma patients, and we show that cfDNA is a biomarker for prognosis and response to treatment.

## 2 Materials and methods

### 2.1 Patients

A prospective longitudinal biomarker cohort study was performed in collaboration between Cancer Research UK Manchester Institute and The Christie NHS Foundation Trust. Ethical approval was granted by the Manchester Cancer Research Centre (MCRC) Biobank Access Committee (Protocol number 13RIMA01). All patients gave written informed consent. Inclusion criteria were the diagnosis of metastatic melanoma, patients naïve for systemic oncological treatments or with an interval from therapy (in the adjuvant or metastatic setting) of at least 2 years, to be longitudinally followed up during treatment. Patients were studied with paired blood collections and scan assessments performed before treatment initiation and at treatment response evaluation (at 12–16 weeks).

### 2.2 Tumour burden and response assessment

Tumour burden estimation was performed with computed tomography (CT), magnetic resonance imaging (MRI) or positron-emission tomography coupled with CT (PET-CT) (slide thickness 3 mm). Scans were evaluated by a radiologist as per RECIST 1.1 and then independently reviewed for metastases volume analysis; Picture Archiving and Communication System Centricity Enterprise Web Viewer (v 3.0, GE Healthcare, Boston, United States of America (US)) was used. Tumour burden was assessed as the sum of all three-dimensional metastatic lesions volumes (calculated with the formula for ellipsoid volumes, as previously described [18]) visualized at radiological scans. The maximum interval between blood biomarker analysis and radiological assessment was 4 weeks at baseline and less than 2 weeks for treatment response follow-ups. The variation of plasma cfDNA and tumour burden at baseline and clinical reassessment was defined as the ratio between cfDNA concentration at reassessment to baseline and the ratio of tumour burden at reassessment to baseline.

### 2.3 Circulating biomarkers

Blood samples were collected in BD Vacutainer K2E (EDTA), plasma was separated and frozen within 4 h of phlebotomy. DNA was extracted from plasma using QIAamp Circulating Nucleic Acid Kits (Qiagen). CfDNA was quantified by means of measurement of a reference gene, RNAseP, by quantitative polymerase chain reaction (PCR) using the TaqMan RNAseP Assay (Life Technologies); in 33 patients, ctDNA was additionally quantified by means of droplet digital PCR Bio-Rad QX200 targeted to a specific mutation in melanoma cells and expressed as variant allele frequency (VAF), the fraction of mutant droplets among the total number of mutant and wild-type droplets, as previously described [17], [18]. Lactic dehydrogenase (LDH), glomerular filtration rate (GFR) (calculated with Cockroft-Gault formula [21]) and WBC count were measured during routine clinical diagnostics.

### 2.4 Statistical analyses

**Fig. S1:**
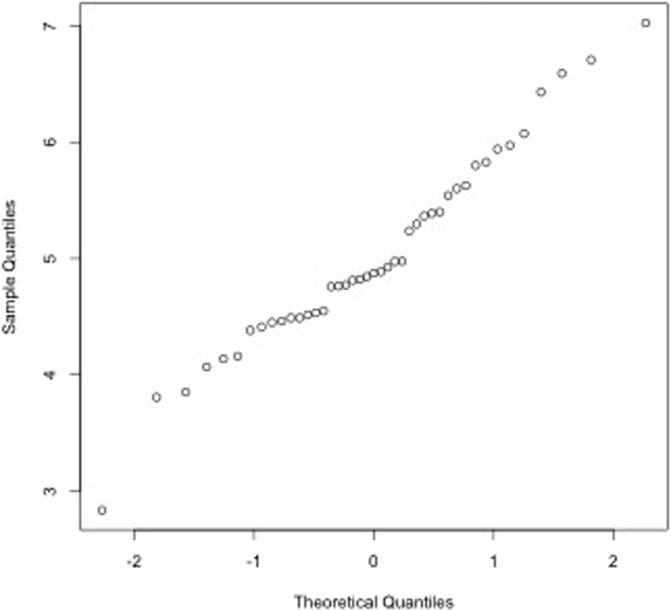
**Distribution of plasma cell-free DNA in melanoma patients.** The deviations from the straight line of the Q–Q plot are minimal, confirming the likelihood of a normal distribution.

After checking the assumptions of normality distribution (Anderson-Darling test A = 0.36, *P* = 0.425, where a *P* value < 0.05 would reject the hypothesis of normality; Supplementary Fig. 1 shows the Q–Q plot for the normal distribution test), Pearson test was used to assess a pairwise correlation between cfDNA and tumour burden both at baseline and at treatment response reassessment (minimum sample size to identify a ρ value > 0.45, with α = 0.05 and β = 0.8 was calculated 36 patients) and between cfDNA, GFR and WBC. The multivariate analysis for the relationship between cfDNA and tumour volume, WBC and GFR was performed with linear regression (Cohen rule was used to calculate the sample size with minimum of 34 cases for 3 independent covariates to be tested; f^2^ = 0.35).

Kaplan–Meier method was used for survival analysis; groups were compared with log-rank test. Analysis of HR was performed with multivariate Cox regression and ANOVA to determine the significance level; proportional hazard hypothesis was verified by Schoenfeld residuals test resulting *P* = 0.984, where *P*<0.05 would indicate a violation of the hypothesis residuals and strata for treatment were applied. The ‘rule of the thumb’ was used to calculate minimum size of 20 events for the prognostic study. Receiver Operating Curve (ROC) was used to identify the cut off of baseline cfDNA concentration for overall survival less than one year.

Enrolment continued to fulfillment of sample size for all study parts. All patients were included in the prognostic study. 43 patients were included in the baseline biomarkers study; patients with paired assessments at baseline and first re-assessment were included in the biomarker study for tumour burden and treatment response if the treatment was not stopped or changed in the meantime, for example because of clinical progression or toxicity, (*N* = 38).

Analyses were performed using R (v. 3.3.3, CRAN project, Vienna).

## 3 Results

**Fig. 1:**
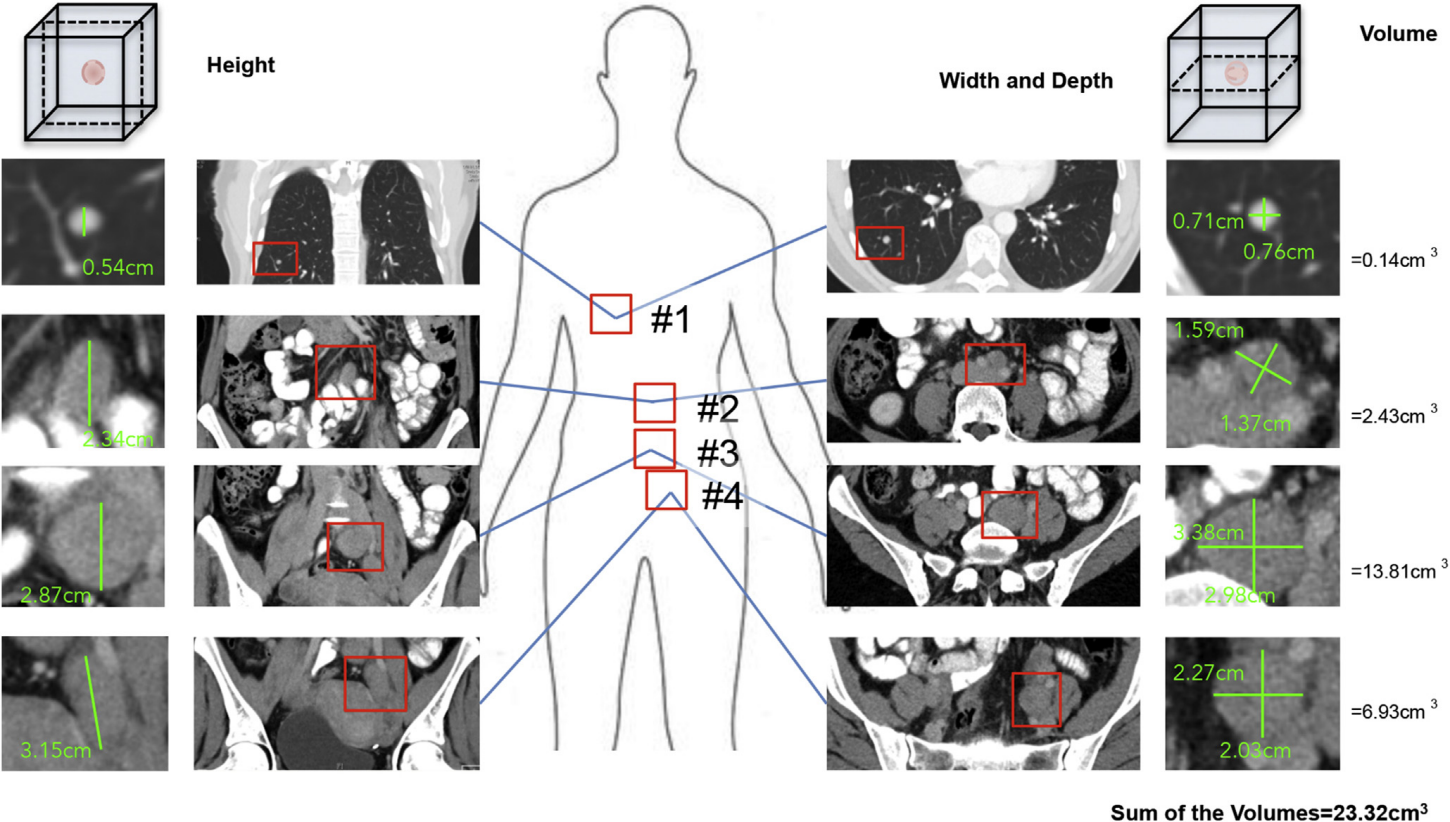
**Method to measure total tumour burden in melanoma patients**. The figure shows the procedure to determine total tumour burden in an individual patient (patient #36). The scan images of the metastases on the frontal (measuring the height of the metastases) and axial plane (measuring the width and depth of the metastases) are reported on the left and right, respectively. Correspondent magnified images are on the far left (frontal plane) and far right (axial plane). In descending order, patient #36 had a lung metastasis (#1), a peri-aortic nodal metastasis (#2), a right peri-common iliac nodal metastasis (#3) and a right peri-bifurcation iliac nodal metastasis (#4). Each metastasis was measured in its 3 dimensional orthogonal diameters (A, B and C in the formula below) and then the volumes, calculated with the formula for ellipsoids (Volume = (diameter A/2) × (diameter B/2) × (diameter C/2) × π4/3), shown for each metastasis on the right side of the figure, and then summed.

To investigate the relationship between tumour burden and the levels of total cfDNA in plasma, we first measured the cumulative volume of all the metastatic lesions in 38 melanoma patients attending the Christie Hospital NHS Foundation Trust. To determine tumour burden, CT or MRI scans were used to identify all of the lesions in each individual patient, and then we measured the height, width and depth of each lesion (Fig. 1). These measurements were used to calculate the volume of the single lesions, and were then summed to give the tumour burden for each patient (Fig. 1). Thirty-eight patients who had received baseline and reassessment scans were included in the study, and an additional 5 patients, whose scans were outside the inclusion criteria but for whom we had cfDNA measurements, were also included in the prognostic analysis. All of the patients had metastatic melanoma, mostly (76%) with stage M1c disease, the median age was 58 (range 18–85), the distribution of men and women was roughly equal, and 37% had a raised LDH (Table 1, Table 2). The mutation status was studied for driver oncogenes with therapeutic potential (*BRAF*, *NRAS*, *KIT*) in this patient population, and the treatment modalities administered reflect the standard of care for patients at that time. The median progression free survival was 4.8 months (range 1.1–15.3) and the median overall survival was 17.3 months (range 2.9–28.3) (Table 1) with a median follow-up time of 11.9 months. 24 patients have died. The majority (76%) of the patients presented with primary melanoma in the skin and subsequently developed multi-organ metastatic disease (79%). The number of metastatic sites ranged from 1 to 7 lesions, most commonly in the lymph nodes (71% of cases), but also in other sites frequently associated with metastatic melanoma. The tumour burden across the cohort ranged from 0.45 cm^3^ to 310 cm^3^, with a median of 20.85 cm^3^ (Table 1, Table 2).

**Table 1:** Patient demographics and disease characteristics.

Patient characteristic	Median (range) tot = 43	*N* (%) tot = 43
Sex F:M		18(42):25(58)
Age (years)	58.1 (18–85.1)	
Subtype skin:mucosal:undetermined		37(86):3(7):3(7)
LDH (U/L; upper normal limit = 550)	467 (268–1780)	
BRAF mutation		30 (70)
NRAS mutation		7 (16)
cKIT mutation		1 (2)
No detected mutations (‘wild type’)		5 (12)
Treatment		
ipilimumab		15 (35)
BRAF/MEK inhibitor		14 (33)
PD1 inhibitor		11 (26)
DTIC		1 (2)
Progression free survival (months)	4.8 (1.1–15.3)	
Overall survival (months)	17.3 (2.8–28.3)	
BRAF-mutated overall survival (months)	10.4 (2.9–19)	
NRAS-mutated overall survival (months)	18.8 (3.8–18.8)	
KIT-mutated overall survival (months)	27.9 (NA)	
WT overall survival (months)	22.7 (7.6–22.7)	
1-year survival (%)		55.9

DTIC, dacarbazine; LDH, lactic dehydrogenase; cfDNA, cell free DNA.

**Table 2:** Melanoma characteristics and stage of patients analysed for tumour burden.

Patient	Stage	Baseline LDH	Number of metastatic sites	Metastatic sites	Baseline tumour burden (cm^3^)	Baseline cfDNA (pg/μl)	1st line therapy	OS (months)
#1	M1c	high	2	lung, nodes	2.74	213.8	ipilimumab	23
#2	M1a	low	1	nodes	115.98	80	ipilimumab	3.8+
#3	M1c	low	2	liver, nodes	5.89	86.6	DTIC	27.9+
#4	M1b	low	3	lung, nodes, soft tissues	3.42	17	ipilimumab	22.7+
#5	M1c	low	3	lung, nodes, bone	24.25	89	ipilimumab	7.6+
#6	M1c	high	1	nodes	95.17	255	BRAFi	4.6+
#7	M1c	low	2	lung, liver	4.98	117	BRAFi	17.3+
#8	M1c	low	2	liver, peritoneal	3.55	44.9	ipilimumab	18
#9	M1c	low	2	nodes, bowel	4.78	58.35	ipilimumab	28.3
#10	M1c	low	3	liver, nodes, soft tissues	7.25	124	ipilimumab	10.4+
#11	M1c	high	2	liver, peritoneal	0.45	137.7	BRAFi	2.9+
#12	M1c	low	3	adrenal, peritoneal, soft tissues	25.69	64	ipilimumab	18.8+
#13	M1c	high	3	nodes, bone, bowel	2.74	392.7	PD1i	13.4
#14	M1a	low	1	nodes	28.94	82.4	ipilimumab	23.8
#15	M1c	low	3	liver, nodes, soft tissues	36.64	188	ipilimumab	8.4+
#16	M1c	low	2	nodes, bone	45.15	93	ipilimumab	11.6+
#17	M1c	high	4	liver, peritoneal, retroperitoneal, soft tissues	310.36	1125.3	BRAFi	5.6+
#18	M1c	low	3	liver, nodes, soft tissues	11.79	130.8	PD1i	12
#19	M1c	high	4	lung, liver, nodes, bone	74.78	89	ipilimumab	8.3+
#20	M1a	low	1	soft tissues	25.34	118	ipilimumab	13.2+
#21	M1c	high	7	lung, liver, nodes, bone, adrenal, omental, soft tissue	16.68	729.9	BRAFi	8.1+
#22	M1c	low	2	lung, bone	75.83	278	BRAFi	8.7+
#23	M1a	low	2	lung, nodes	68.81	144.9	ipilimumab	15.7
#24	M1a	low	1	nodes	4.49	270.8	PD1i	14.6
#25	M1c	high	1	brain	6.96	819.3	BRAFi	8.5+
#26	M1c	low	3	lung,liver, soft tissues	3.03	199.6	PD1i	14.2
#27	M1a	low	1	nodes	13.72	221.2	PD1i	11.3
#28	M1c	low	2	peritoneal, soft tissues	1.06	47	PD1i	11.9
#29	M1c	high	4	lung, liver, nodes, bone	24.97	621	BRAFi	6.8+
#30	M1b	low	2	lung, nodes	22.94	379.9	PD1i	10.4
#31	M1c	low	4	lung, liver, nodes, bone	2.75	91.4	PD1i	10
#32	M1c	high	3	lung, nodes, soft tissues	62.28	339.8	BRAFi	19+
#33	M1c	low	2	nodes, soft tissues	48.24	330.3	BRAFi	4.9+
#34	M1c	low	1	nodes	12.08	132.3	BRAFi	10.2
#35	M1b	low	2	lung, nodes	32.43	62.6	PD1i	9.5
#36	M1c	low	3	lung, liver nodes, soft tissues	23.32	144.6	BRAFi	8.2
#37	M1c	high	2	brain, nodes	18.75	218.8	BRAFi	8.5
#38	M1c	high	1	lung	26.98	94.5	PD1i	7.4
							ipilimumab	23
median			2 (1–7)		20.85 (0.45–310.36)	135 (17–1125.3)		

DTIC: dacarbazine; BRAFi: BRAF-inhibitor based therapy; PD1i: PD1 inhibitor; +: dead.

**Fig. 2:**
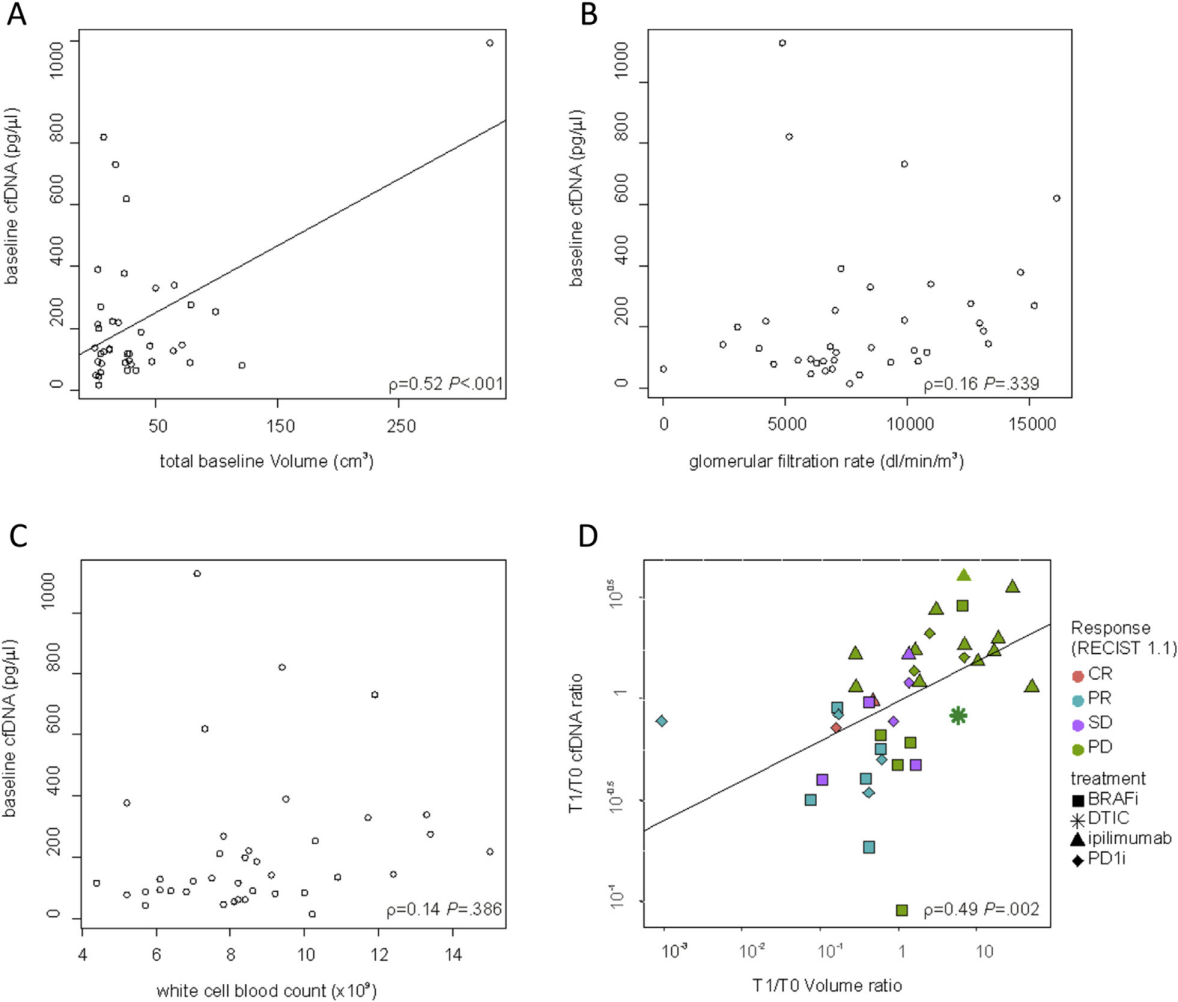
**Plasma cfDNA is a biomarker for tumour burden in metastatic melanoma**. **(A**–**C)** Scatter plots, showing the correlation between baseline cfDNA concentration and pre-treatment tumour burden (A), renal glomerular filtration rate (B) and white cell count (C). Each dot represents the value of a single patient; slanting lines are the regression lines. (D) Scatter plot showing the correlation between the variations (the ratio between values at first re-assessment for treatment response and baseline) of cfDNA concentration and tumour burden. Each dot is representative of the value of every single patient, slanting lines is the regression line. CR: complete response; PR: partial response; SD: stable disease; PD: progressive disease; BRAFi: BRAF-inhibitors based therapy; DTIC: dacarbazine; PD1i: PD1 inhibitors.

**Fig. S2:**
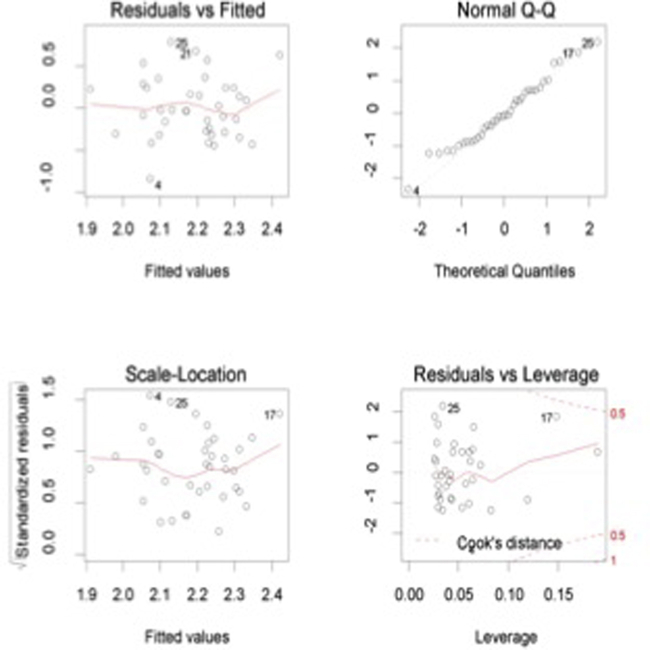
**Survival analysis.** Kaplan–Meier curves showing overall survival (OS) of patients relative to baseline cfDNA concentration quartiles. The continuous line presents patients with baseline cfDNA in the lower quartile (<89 pg/μL, *N* = 10), the dashed line the combined central quartiles (≥89 pg/μl and <262.9 pg/μL, *N* = 22), and the dotted line the upper quartile (≥262.9 pg/μL *N* = 11).

We measured baseline total cfDNA in the patients' plasma, and observed a range from 17 pg/μL to 1.125 pg/μL with a median of 135 pg/μL (Table 2). We found a significant correlation (ρ = 0.52, *P* < 0.001) between baseline plasma total cfDNA and pre-treatment tumour burden (Fig. 2A). In contrast, cfDNA levels did not correlate with GFR (ρ = 0.16, *P* *=* 0.339) (Fig. 2B) or WBC count (ρ = 0.14, *P* *=* 0.386) (Fig. 2C). Multiple regression analysis yielded similar results, confirming cfDNA concentration correlated with tumour burden (R^2^ = 0.36, *P* < 0.001), but not glomerular filtration rate or white blood cell count (*P* = 0.135 and *P* = 0.080 respectively) (the validation of the multiple regression is shown in Supplemental Fig. S2).

We also compared the relationship between the change in plasma cfDNA and change in tumour burden between baseline and first response assessment (∼3 months for immunotherapy and ∼4 months for targeted therapy) and observed a significant correlation (ρ = 0.49, *P* = 0.002). Note, the correlation was consistent across all treatment types (Fig. 2D). Thus, the correlation between cfDNA and tumour burden was maintained after treatment. Of note, one of the patients presented only brain metastases, and even for this patient there was a correlation between cfDNA at baseline (819.3 pg/μL) and following a partial response to dabrafenib (130.8 pg/μL).

**Fig. 3:**
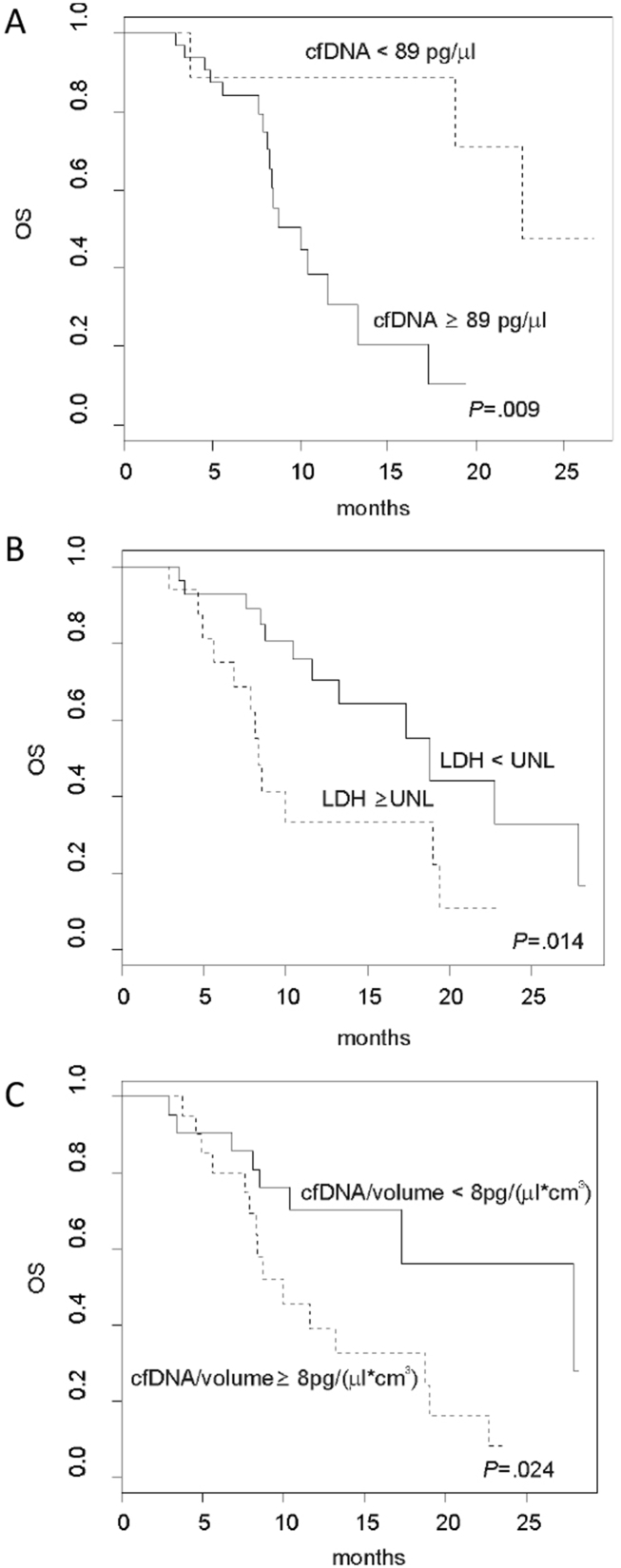
**Plasma cfDNA is prognostic in metastatic melanoma patients**. **(A**–**C)** Kaplan–Meier plots for melanoma patients showing overall survival (OS) relative to baseline plasma total cfDNA concentrations (A), plasma LDH levels (B) and the ratio between cfDNA and tumour burden (C). In (A) the continuous line represents patients with low baseline cfDNA concentration (<89 pg/μL, *N* = 10), the dashed line the high baseline cfDNA cohort (≥89 pg/μl, *N* = 33). In (B) the continuous line represents patients with low baseline LDH value (*N* = 27) and the dashed line patients with high baseline LDH (*N* = 16). In (C) the continuous line represents patients with low baseline cfDNA shedding coefficient (*N* = 19) and the dashed line patients with high cfDNA shedding coefficient (*N* = 19).

**Fig. S3:**
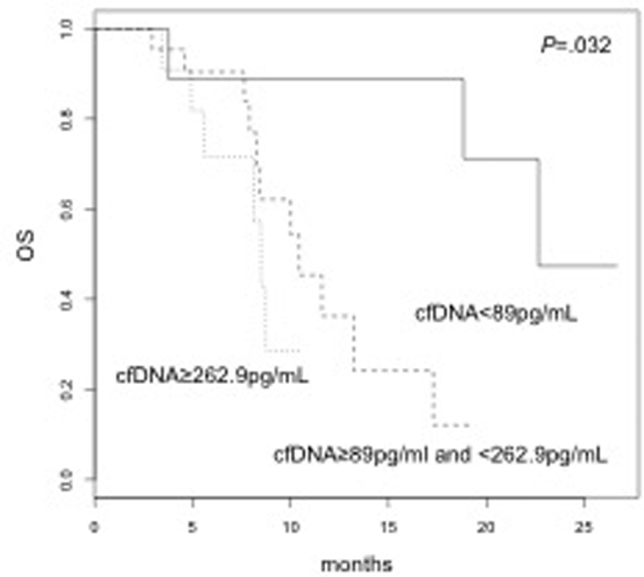
**Determining operational limits for cfDNA concentration and cfDNA shedding. (A, B)** Receiver Operating Curve (ROC) was used to identify the best cut-off to discriminate the worse prognosis (overall survival less than one year) patients for baseline plasma cfDNA concentration (A) and cfDNA shedding coefficient (B).

**Fig. S4:**
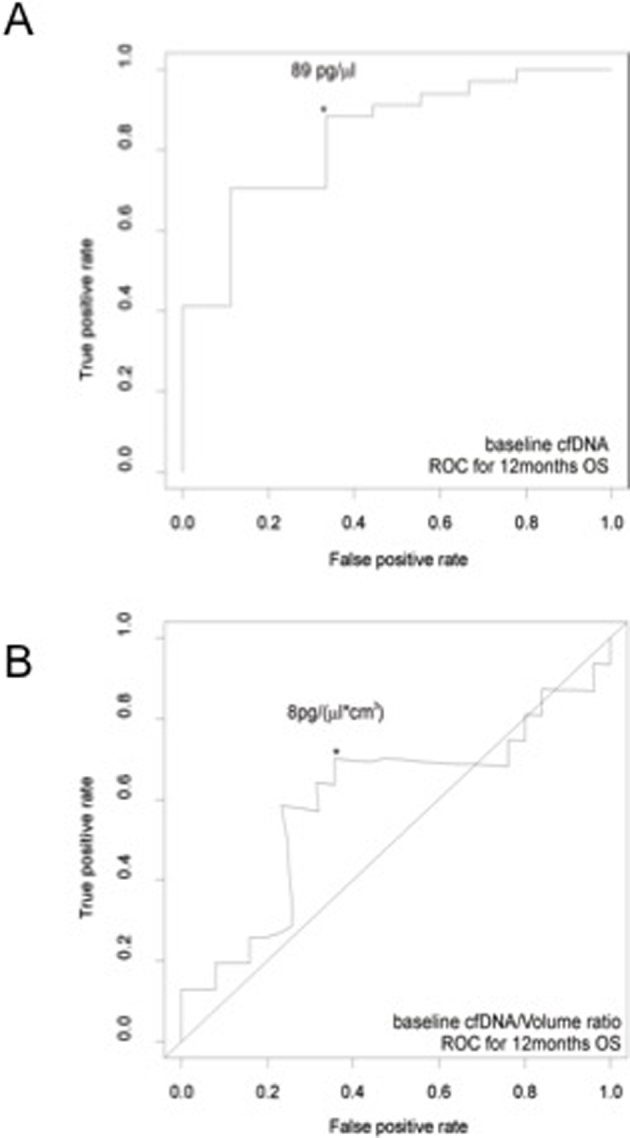
**Validation of the linear regression multivariate analysis of baseline cfDNA, tumour burden, renal clearance and while cell count values.** Validation plots for the multivariate linear regression model used to analyse the relationship of baseline cfDNA, tumour burden, glomerular filtration rate and while cell count values.

We performed survival analysis by comparing the patients with baseline cfDNA in the lower quartile (<89 pg/μL, *N* = 10), aggregated central quartiles (≥89 pg/μl and <262.9 pg/μL, *N* = 22) and upper quartile (≥262.9 pg/μL *N* = 11), including all 43 patients in this analysis. We found that overall survival differed if cfDNA was low (22.7 months), intermediate (10.4 months) or high (8.5 months; *P* = 0.032) (Supplementary Fig. S3). To facilitate practical application of our findings, we determined the optimal baseline cfDNA concentration cut-off for survival shorter than one year using a ROC-determined value of 89 pg/μL (corresponding to the lower quartile limit; area under the curve = 0.84) (Supplementary Fig. S4A). Above this cut-off, patients presented a median overall survival of 10.0 months, whereas below this cut-off, patients presented an overall survival of 22.7 (*P* = 0.009; Fig. 3A). Similarly, we analyzed serum LDH, the standard prognostic biomarker for melanoma, and found that patients with LDH above the upper normal limit (550U/L) had a worse prognosis (8.3 versus 18.8 months, *P* = 0.014) (Fig. 3B).

Next, we performed multivariate Cox regression analysis to determine if cfDNA or LDH is the better prognostic factor in our patients. Firstly we analyzed cfDNA and LDH as continuous covariates without bias produced by grouping and found that cfDNA was significantly associated with the hazard of death (for additional unit increase of cfDNA HR (95% CI) = 1.002 [1.001–1.004]; *P* = 0.009) but LDH was not (for additional unit increase of LDH, HR (95% CI) = 1.001 (1–1.002); 0.277). We then performed a multivariate Cox regression analysis using cfDNA and LDH as categorical variables (high versus. low, using the cut-off of 89 pg/μL and the upper normal limit, respectively) and cfDNA was confirmed to be a better prognostic factor (HR = 2.22 for patients with cfDNA concentration ≥89 pg/μL; 95% CI 1.18–5.55, *P* = 0.004) (Supplementary Fig. S4B), than LDH (HR = 1.9 for patients with LDH ≥ upper normal limit; 95% CI 1–7.9, *P* = 0.213).

We next examined if the ratio of plasma cfDNA to tumour volume (the cfDNA shedding coefficient, defined as the ratio of cfDNA concentration in plasma to tumour burden) impacted on prognosis. To assess if patients with similar tumour burden had a worse outcome if their baseline plasma cfDNA level was relatively higher. We identified 8 pg/(μL*cm^3^) as the optimal cut-off of the shedding coefficient for survival shorter than one year (Supplementary Fig. 4B) and observed that patients with a high cfDNA shedding coefficient (cfDNA/tumour volume≥8 pg/[μL*cm^3^]) had worse prognosis than those with a low cfDNA shedding coefficient (HR = 2.7, 95% CI 1.1–6.7, *P* = 0.029), with a median overall survival of 11.6 months and 27.9 months respectively (*P* = 0.024) (Fig. 3C).

**Fig. 4:**
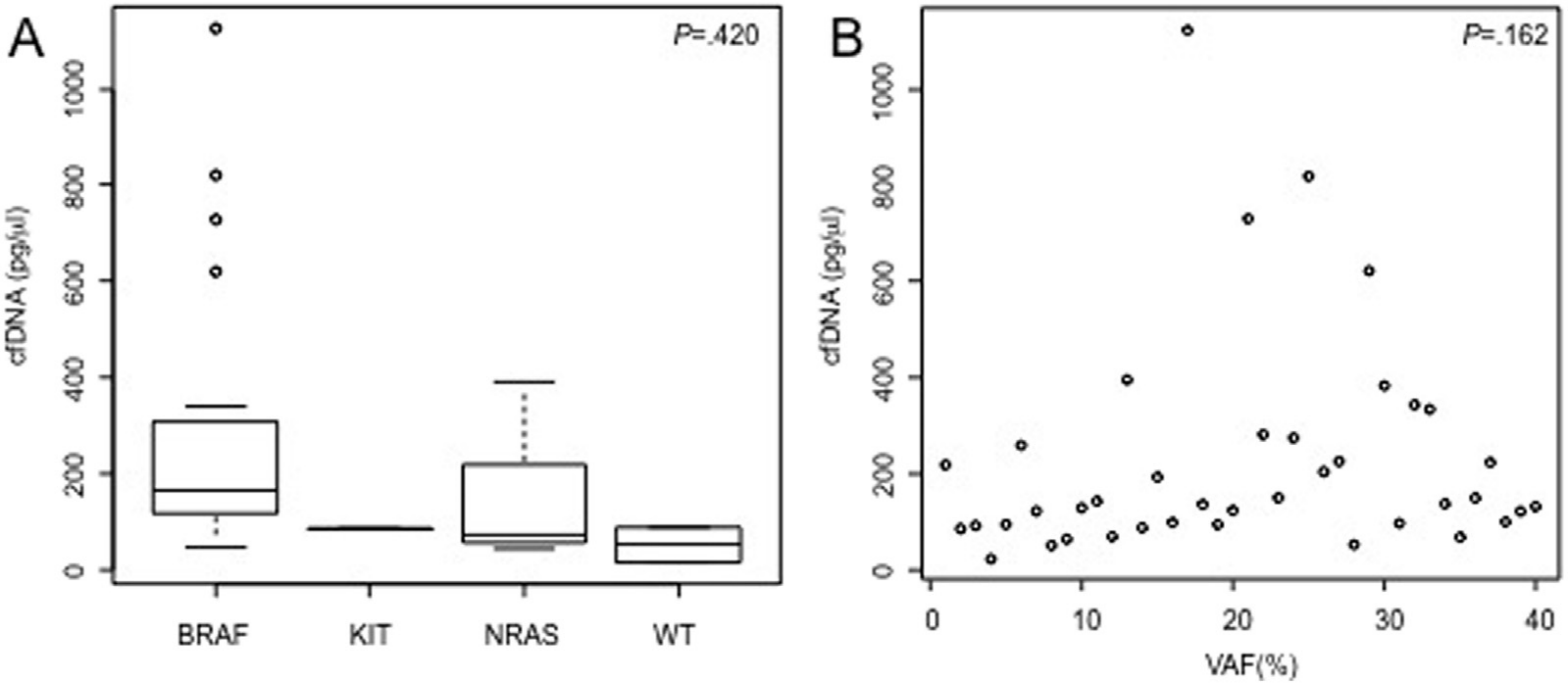
**Plasma cfDNA is independent of driver oncogene mutation status. (A)** Bars show the cfDNA concentration for metastatic melanoma patients according to mutational status of the disease (*BRAF* mutated, *NRAS* mutated, *KIT* mutated or wild type for all these oncogenes). (B) Scatter plot showing the correlation between cfDNA concentration and the variant allele frequency for *BRAF*, *NRAS* or *KIT* mutation measured in plasma of metastatic melanoma patients. Each dot represents the values of a single patient.

Finally, we compared the plasma cfDNA levels in patients whose tumours presented with *BRAF*, *NRAS* or *KIT* mutations, and also in tumours that were wild type for all of the three genes, but did not find any correlation between plasma total cfDNA concentration and mutation group for these therapeutically important driver oncogenes (F value 1.4, 3 degrees of freedom [DF], *P* = 0.254) (Fig. 4A). In addition, to determine if there was a correlation between the plasma variant allele frequency (VAF) for these specific driver oncogenes (ctDNA) and the cfDNA in the patients' plasma, we compared VAF for *BRAF*, *NRAS* and *KIT* to total cfDNA, and found that there was no correlation between these two values (ρ = 0.16, *P* = 0.162) (Fig. 4B).

## 4 Discussion

In this study we demonstrated that there is a correlation between plasma total cfDNA and tumour burden in melanoma patients. By longitudinal sampling, we further showed that the correlation between cfDNA and tumour burden reflected the changes in tumour volumes that occurred with therapeutic intervention. Critically, we showed that baseline levels of total cfDNA were prognostic for OS, and that patients whose tumours released higher concentrations of cfDNA per unit volume had a worse prognosis. Thus, we concluded that plasma total cfDNA is a reliable surrogate biomarker for tumour burden and for changes that occur in tumour burden during treatment. We also showed that in a patient who presented only brain lesions, total cfDNA reflected response to treatment, demonstrating that metastatic sites in the central nervous system also contribute to total ctDNA in the plasma.

Intriguingly, we did not observe a correlation between total cfDNA and individual mutant driver oncogene DNA levels (VAF) in the plasma. Notably, tumour mass visualized by radiological investigations contains not only cancer cells carrying specific driver mutations, but also a vast array of stromal and inflammatory cells that do not harbour those specific mutations. Both cell populations are in constant turnover and are likely to contribute disproportionally to the cfDNA in the blood. Moreover, the proportion of cancer cells to stromal cells within individual tumours will be different, further affecting the amount of specific mutant DNA within the total ctDNA for an individual patient. Furthermore, there is considerable clonal variation between the cancer cells within an individual tumour, and the evolutionary selective pressure imposed by treatment and immuno-surveillance can increase or decrease the cancer cell clonality within disease localization, again affecting the proportion of specific mutant to total ctDNA in the blood.

One of the limits of the present study is the relatively small sample size, allowing the analysis of only two covariates in the prognostic study. A larger number of patients would be needed to include more prognostic factors for melanoma. Furthermore, treatment options for melanoma continue to improve over time, and whether our findings remain valid with emerging treatment options is unknown.

LDH is a well-established prognostic factor in advanced melanoma, though why this is, is not clearly understood. LDH level is routinely considered when advising on treatment options and likely outcome, and has maintained its relevance in the revised AJCC Staging System for melanoma [22]. When compared with cfDNA prognostic value, LDH was not significant at multivariate analysis. In contrast, despite the limited sample size, we demonstrated that cfDNA was prognostic for overall survival. If confirmed, this could lead to cfDNA being established as a biomarker for prognosis stratification and clinical decisions for metastatic melanoma, and not being restricted to a specific mutation could be used independent of genetic subtype. We also identified a cut off baseline cfDNA concentration that could stratify patients for a worse prognosis. Although cut-off values are usually dependent on experimental condition, our identified limit nevertheless allowed us to endorse the prognostic value of cfDNA by showing that patients with cfDNA levels below the cut-off lived longer than patients with values above.

## 5 Conclusions

In conclusion we show that cfDNA should be considered as a prognostic factor and a biomarker that could be used as a surrogate for tumour burden and prognosis with treatment in melanoma patients.

## Role of the funding source

This work was supported by the Wellcome Trust [100282/Z/12/Z] and the Cancer Research UK Manchester Institute [C5759/A12328]. SV was supported by the ESMO Clinical Research Fellowship with the aid of a grant from Novartis. Any views, opinions, findings, conclusions, or recommendations expressed in this material are those solely of the author(s) and do not necessarily reflect those of the Wellcome Trust, the Cancer Research UK Manchester Institute, ESMO or Novartis.

## Disclosures

The authors do not have any conflicts of interest.
